# Novel multi-objective affinity approach allows to identify pH-specific *μ*-opioid receptor agonists

**DOI:** 10.1186/s13321-023-00746-4

**Published:** 2023-09-19

**Authors:** Christopher Secker, Konstantin Fackeldey, Marcus Weber, Sourav Ray, Christoph Gorgulla, Christof Schütte

**Affiliations:** 1 Zuse Institute Berlin, Berlin, Germany; 2 Max Delbrück Center for Molecular Medicine, Berlin, Germany; 3 Institute of Mathematics, Technical University Berlin, Berlin, Germany; 4 Department of Physics, Harvard University, Cambridge, MA 02138 USA; 5 Department of Biological Chemistry and Molecular Pharmacology, Harvard Medical School, Boston, MA 02115 USA; 6 Department of Cancer Biology, Dana-Farber Cancer Institute, Boston, MA 02215 USA; 7 Department of Structural Biology, St. Jude Children’s Research Hospital, Memphis, TN, USA; 8 Mathematics Institute, Freie Universität Berlin, Berlin, Germany

## Abstract

Opioids are essential pharmaceuticals due to their analgesic properties, however, lethal side effects, addiction, and opioid tolerance are extremely challenging. The development of novel molecules targeting the $\mu$-opioid receptor (MOR) in inflamed, but not in healthy tissue, could significantly reduce these unwanted effects. Finding such novel molecules can be achieved by *maximizing* the binding affinity to the MOR at acidic pH while *minimizing* it at neutral pH, thus combining two conflicting objectives. Here, this *multi-objective optimal affinity approach* is presented, together with a virtual drug discovery pipeline for its practical implementation. When applied to finding pH-specific drug candidates, it combines protonation state-dependent structure and ligand preparation with high-throughput virtual screening. We employ this pipeline to characterize a set of MOR agonists identifying a morphine-like opioid derivative with higher predicted binding affinities to the MOR at low pH compared to neutral pH. Our results also confirm existing experimental evidence that NFEPP, a previously described fentanyl derivative with reduced side effects, and recently reported $\beta$-fluorofentanyls and -morphines show an increased specificity for the MOR at acidic pH when compared to fentanyl and morphine. We further applied our approach to screen a >50K ligand library identifying novel molecules with pH-specific predicted binding affinities to the MOR. The presented differential docking pipeline can be applied to perform multi-objective affinity optimization to identify safer and more specific drug candidates at large scale.

## Introduction

The discovery of opium to treat severe pain has been one of the most important achievements in ancient medicine, although manufactured opium preparations in later history had often led to treatment failures due to the fluctuating concentration of the active ingredient. Friedrich Sertürner conducted analytical experiments which in 1804/1805 led to the discovery of the active ingredient of opium: morphine [1]. The pioneering discovery of morphine paved the way for modern drug development. For the first time, the exact dosing of a single active ingredient - and thus a calculable effect on the patient - became possible. Unfortunately, Sertürer became addicted to morphine due to his self-experimentation and ultimately died of his own discovery.

Morphine has lethal side effects. Most notably, it can cause respiratory depression. In order to reduce lethal effects and addiction, Bayer developed heroin with good hope: It is a derivative of morphine which needs a much smaller dose for the same pain relief effect. Heroin, first produced in 1898, was considered a legal drug for pain, cough and other ailments, and of course children were treated with it, too [2]. However, it quickly turned out that heroin is actually more addictive than morphine. The simple adage “the dose makes the poison” has not been a good rule of thumb in this case.

In view of this history, it is surprising why the paradigm of modern drug development, especially when looking for a lead compound (similar to how heroin was a lead compound), still optimizes towards the strongest possible effect, i.e. the lowest possible dose of active ingredient to be administered. In this questionable spirit, a strong affinity of the searched-for lead compound with regard to its target protein is the main goal of current drug screening approaches. In the case of pain relief, the target protein addressed by the active ingredients is the $\mu$-opiod receptor (MOR). In the most simple model (for an *in vitro* experiment) let [*P*] be the equilibrium concentration of free target protein, [*L*] the respective concentration of unbound ligand (opioid), and [*PL*] the concentration of opioids which are bound to the MOR in equilibrium. The binding process can be expressed by a reaction equation

$$\begin{aligned} P + L \mathop {\rightleftharpoons }\limits ^{k_{ass}}_{k_{diss}} PL \end{aligned}$$

with association and dissociation rates. The affinity of the ligand with regard to the receptor is then defined as the ratio

$$\begin{aligned} \text {Affinity}_P(L)=\frac{k_\text {ass}}{k_\text {diss}}=\frac{[PL]}{[P][L]}. \end{aligned}$$

This is usually the quantity to be maximized when looking for an “optimal” lead compound in conventional drug screening.

Having identified a target protein, virtual drug screening methods can be used to find ligands with high affinity to the target and are becoming increasingly important for efficient drug discovery [3, 4]. In order to estimate the affinity for experimentally untested pairs of ligands and target proteins, there exist some heuristic approaches. Instead of analyzing or computationally modeling the binding process itself in order to derive the rate constants, often only the three-dimensional placement of the ligand inside the target protein (docking) is optimized using a so-called scoring function *f*. This function *f* depends on the coordinates of the atoms of the ligand molecule with regard to the atomic coordinates of the respective structure *T*(*P*) of the target protein. Estimating the affinity of a given ligand to a target protein is equivalent to finding the global minimum of a scoring function

$$\begin{aligned} \text {Affinity}_P(L)= \min _{\text {coords}(L)}f\left( \text {coords}(L),\text {coords}(T(P))\right) , \end{aligned}$$

where the “=” sign means equality up to the approximations made. In the *optimal affinity approach* one then aims to find ligands $L^*$ maximizing the affinity, that is, for which

1 $$\begin{aligned} \text {Affinity}_P(L^*) = \max _L \text {Affinity}_P(L). \end{aligned}$$

Since one often is not only interested in the optimal ligands but in a list of promising candidate ligands, one searches for ligands $L^*$ such that

2 $$\begin{aligned} \text {Affinity}_P(L^*)\ge (1-\theta )\; \max _L \text {Affinity}_P(L), \end{aligned}$$

where $0\le \theta \ll 1$ denotes a small parameter allowing for a minor relative deviation from the maximum.

One of the most often used scoring functions is given by the (Gibbs) free energy difference $\Delta F_P(L)$ between the unbound and the bound state of the ligand-protein system. In this case, the scoring function is given by $f=\exp (-\beta |\Delta F_P|)$ with a system-dependent constant $\beta$ such that ligands *L* that minimize *f* are the ones that maximize the free energy difference $|\Delta F_P|$. Then, the optimization criterion (1) is replaced by

3 $$\begin{aligned} |\Delta F_P(L^*)| = \max _L |\Delta F_P(L)|. \end{aligned}$$

The fact that the set of all chemically possible ligands includes about $10^{60}$ structures makes it - even with nowadays increased computer power - impossible to explore this huge “chemical space” (e.g. [5–7]). Several methods to systematically cope with this problem have been developed by either advanced search strategies or by confining the search space. Among the search strategies are fragment-based methods. In these methods initial ligands are cut into rotatable or scalable fragments and these fragments are then fitted into the binding pocket [8–11]. By these methods, not all molecules of the chemical space are screened but only those that geometrically fit into the pocket. Also, mathematically motivated search strategies such as (local and global) Monte-Carlo methods [12], multi-agent/ swarm intelligence-based methods [13] or genetic algorithm-based methods [14] can be found. Aside from these search strategies also different scoring functions are possible.

The optimal affinity approach, however, does not lead to a solution to the problem of finding promising drug candidates with minimal side effects because large affinity screenings in the first place do not take additional constraints into account. The nowadays widely used opioid fentanyl - discovered in the 1960s - shows strong (almost optimal) affinity to the MOR, and it is even more potent and cheaper to produce than heroin. However, it has the same lethal side effects and fentanyl is one of the causes of the opioid crisis in the USA [15]. Due to this crisis, there is a strong need to find better strong pain-relieving drugs, which do not show severe side effects.

Summing up, the conventional drug screening approaches for finding a new lead compound are mainly based on maximizing the binding affinity of the protein-ligand system. However, also other factors can influence the efficacy. We therefore propose a novel approach, broadening this view:

Instead of investigating $\text {Affinity}_P(L)$ we seek for novel lead compounds with respect to

$$\begin{aligned} \text {Affinity}_P(L,\mathcal{E}), \end{aligned}$$

where $\mathcal E$ represents the chemical environment of the system.

In the literature, several cases have been documented, where the environmental conditions can have an influence on the protein-ligand system. For instance, in [16] it has been observed, that the binding properties of the human serum albumin, which is critical for drug half-life and distribution, change with respect to the surrounding temperature. But also the composition of the surrounding solvent and its solubility can influence the binding affinity (e.g. [17]). In addition, other factors like the pH value of the environment can influence the binding affinity.

For example, in the context of the above-mentioned fentanyl, we previously exploited the surrounding pH value of the target protein to design a novel drug: Here, the wanted effect (pain relief), as well as the unwanted, potentially lethal effects, are both caused by binding of the opioid to the MOR. A primarily high binding affinity (thus, reducing the needed dose) of the searched-for ligand with regard to the MOR is therefore *not* the solution. One solution could be given by the following new paradigm: The pain relief effect can be caused by an opioid that binds to the MOR in *inflamed tissue*, whereas the lethal side effects are primarily caused by opioids that bind to MOR in *healthy tissue*. The difference between inflamed and healthy tissue is e.g. given by the pH value of the chemical environment of the MOR. Inflamed tissue has a low (acidic) pH, healthy tissue has a neutral pH value. The three-dimensional structure of the MOR has a different conformation at low pH than at neutral pH, i.e., the target structure also depends on pH. And also the opioid (ligand) may change its chemical properties and structure with changing pH.

On the basis of this knowledge we propose to change the paradigm in (virtual) drug screening by including the effect of the environment already in the primary search for a lead structure. Our approach proposed in this article represents the environment $\mathcal{E}$ by a parameter $\theta$, in this case a scalar parameter given by the pH value, however, in general a parameter vector. Then, the decisive difference in the environment is represented by two parameter values, $\theta _1$ and $\theta _2$, in our case low and, respectively, neutral pH values. The proposed *multi-objective optimal affinity approach* then seeks for ligands $L^*$ that solve the multi-criteria optimization problem

4 $$\begin{aligned} \max _L \begin{bmatrix} \text {Affinity}_{P}(L(\theta _1),T(\theta _1))\\ -\text {Affinity}_{P}(L(\theta _2),T(\theta _2)) \end{bmatrix}, \end{aligned}$$

where $L(\theta _i)$ and $T(\theta _i)$ denote the ligand and target structures of environment parameter $\theta _i$, $i=1,2$. The minus sign in the second row means that we seek to *minimize* the affinity for $\theta _2$ (neutral pH, healthy tissue in our example) while we seek to *maximize* the affinity for $\theta _1$ (low pH, inflamed tissue).

Solving multi-criteria optimization problems means exploring the set of possible ligands that satisfy (4). In general, this set, often called the Pareto set, contains many ligands, in particular all the ones that solve [18]

5 $$\begin{aligned} \max _L \Big [\lambda \cdot \text {Affinity}_{P}(L(\theta _1),T(\theta _1)) -(1-\lambda )\cdot \text {Affinity}_{P}(L(\theta _2),T(\theta _2)) \Big ], \end{aligned}$$

for one of the values of $\lambda$ between 0 and 1. When using the free energy difference as scoring function, we alternatively may replace (5) by

6 $$\begin{aligned} \max _L \Big [\lambda \cdot |\Delta F_{P}(L(\theta _1),T(\theta _1))| -(1-\lambda )\cdot |\Delta F_{P}(L(\theta _2),T(\theta _2))| \Big ],\qquad \lambda \in [0,1]. \end{aligned}$$

**Fig. 1:**
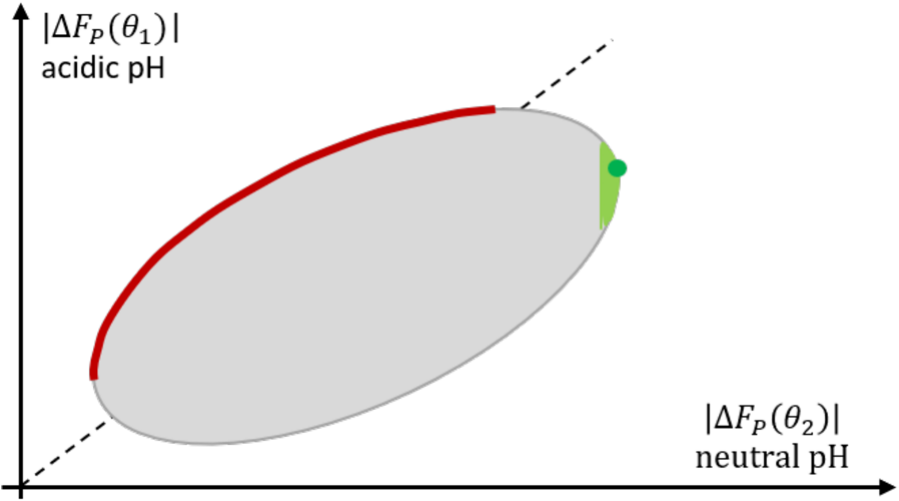
Illustration of the multi-objective optimal affinity approach proposed herein in contrast to the standard optimal affinity approach: Under the assumption that the binding free energy values $F_P(\theta _1)$ and $F_P(\theta _2)$ of all ligands of interest fill the grey shaded area including its boundaries, the standard optimal affinity approach (3) would identify the ligand indicated by the green ball, or, in its relaxed version (2), the green shaded area close to the ball. In contrast, the multi-objective optimal affinity approach (6) would identify the ligands in the red area of the boundary giving higher importance to the binding affinity at acidic pH

The ligands satisfying (6) cannot be improved in any of the two objectives, $|\Delta F_P(\theta _1)|$ and $-|\Delta F_P(\theta _2)|$ without degrading the other objective; these are called Pareto-optimal in the literature on multi-objective optimization. The difference between this proposed multi-objective affinity approach and the standard optimal affinity approach is illustrated in Fig. 1.

In this article, we will not go into the details of how to find algorithms for solving the multi-objective optimization (4) in the entire chemical space; there is an established literature on efficient algorithms, see, e.g., [19], but these algorithms would require extensive specifications for the problem at hand, which is not in the focus of this article. In contrast, we will discuss the new multi-objective optimal affinity approach by exemplifying it for use in virtual drug discovery.

## Methods

**Fig. 2:**
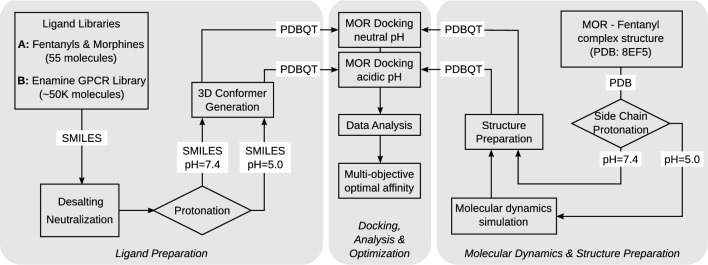
Sketch of the differential docking pipeline for the identification of pH-specific MOR ligands. A ligand library was selected and prepared for docking at neutral pH (7.4) and acidic pH (5.0). Target MOR structures (without the G-protein complex) were derived from an experimentally determined structure (Protein Data Bank [22] (PDB): 8EF5 [23]) and side chain protonation states were generated at pH 7.4 and 5 according to $\hbox {p}K_{\hbox {a}}$ values determined by PROPKA. Docking studies were performed with ligands prepared at pH 7.4 to the neutral MOR (conformation of the experimental structure) and ligands at pH 5.0 to the protonated MOR (conformation after simulation in an acidic environment)

Next, we describe how the *multi-objective optimal affinity approach* can be implemented in practice. We have built a flexible and high-throughput capable differential virtual screening and docking pipeline. The pipeline is general, i.e. it can handle diverse characterizations of the environment by different parameters. However, it is herein explained for our guiding example, the case of MOR-agonists at acidic and neutral pH, see Fig. 2 for a schematic overview. The pipeline is composed of six main steps:

1. *Protonation state generation and Molecular dynamics:* The protonation state of the target structure is generated at neutral and acidic pH using PROPKA [20]. MD simulation is performed to generate the target structure of the MOR at acidic pH. For the neutral environment, the experimentally determined conformation is used (Fig. 2, right panel).
2. *Structure Preparation:* The target structure is prepared for docking and the target space is selected for ligand-target docking.
3. *Ligand Preparation:* After a selection of appropriate ligand libraries, the virtual screening platform VirtualFlow [21] is used to prepare the ligand libraries under pH-specific conditions (Fig. 2, left panel).
4. *Docking:* Using VirtualFlow again, separate, parallelized docking runs are performed (Fig. 2, middle panel), including calculation of the respective binding free energies $\Delta F_P$.
5. *Analysis:* Results of binding free energy calculations are analyzed statistically and the respective binding free energy docking scores are computed.
6. *Optimization*: Pareto-optimal ligands, that is, those that solve the multi-objective optimization problem (6), are identified.

Next, the methods used for the implementation of these six steps are described:

### Step 1: Protonation state generation and molecular dynamics

For molecular modeling, a human MOR structure with fentanyl was procured from the PDB archive [22] (PDB: 8EF5 [23]). The protonation states of the different amino acid residues of the MOR were determined using the PROPKA predictor tool [20, 24, 25], with respect to the system acidity values of pH 5.0 and pH 7.4, corresponding to the inflamed and healthy system states, respectively. The fentanyl was sketched and parameterized using the CHARMM-GUI *Ligand Reader & Modeler* [26].

For docking at neutral pH, the native conformation of the experimental structure (PDB: 8EF5) was used. To generate a target conformation at acidic pH, the protonated MOR-fentanyl complex was inserted into the 1-palmitoyl-2-oleoyl-sn glycerol-3-phosphatidyl choline (POPC) bilayer models using the CHARMM-GUI *Membrane Builder* [27]. Molecular dynamics (MD) simulation was performed with GROMACS 2022.5 [28], using the CHARMM36m force-field for the ligands [29], proteins [30] and lipids [31]. The CHARMM TIP3P water model [32] was used as an explicit solvent. Sodium and chloride counterions were added to neutralize the excess charge and obtain a salt concentration of 0.15 M. The particle mesh Ewald (PME) method [33] was employed to calculate long-range Coulombic interactions, with a 1.2 nm cut-off for real-space interactions. A force-switch function was implemented for the Lennard–Jones interactions, with a smooth cut-off from 1.0 to 1.2 nm. The temperature was maintained at 310 K using the Nosé-Hoover thermostat [34, 35]. System pressure was kept at 1 bar with the Parrinello-Rahman barostat [36] using a semi-isotropic scheme, where pressure along *x*-*y*-directions and the *z*-direction were coupled separately. Coupling constant and compressibility of the barostat were set to 5 ps and $4.5\times 10^{-5}$ bar, respectively. The LINCS algorithm [37] was used to constrain the covalent bonds between hydrogen and other heavy atoms, allowing a simulation time-step of 2 fs.

The simulation system went through consecutive minimization, equilibration, and a production run using the GROMACS scripts generated by the CHARMM-GUI [27]. First, the system was energy minimized with the steepest descent algorithms, followed by six-step equilibration runs. The first two runs were performed in the NVT (constant particle number, volume, and temperature) ensemble, and the remaining runs in the NPT (constant particle number, pressure, and temperature) ensemble. Restraint forces were applied to the fentanyl, MOR, POPC, and water molecules, and *z*-axis positional restraints were placed on POPC atoms to restrict their motion along the *x*-*y*-plane. These restraints were gradually reduced during the equilibration process.

Additional restraints were applied throughout equilibration to keep the distance between the crucial ASP 149$^{3.32}$ and HIS 299$^{6.52}$ residues of the MOR binding site [38, 39] and the fentanyl molecule to the minimum possible. This ensured a similar starting conformation compared to the native structure for the simulation in an acidic environment.

Ultimately, an unrestrained NPT production run of 10 ns was performed, with periodic boundary conditions along all three orthonormal directions. The production run trajectory was saved every 10 ps, and processed with GROMACS analysis tools to generate the required information.

For Root Mean Square Deviation (RMSD) based conformational clustering, the last 50 trajectory frames (0.5 ns) from the production run were considered. The heavy (non-hydrogen) atoms of fentanyl were taken as a reference for the RMSD calculation and subsequent clustering with the *gmx cluster* tool using the “gromos” algorithm [40]. Based on the cluster number and population, an RMSD cut-off of 0.05 nm was chosen for selecting the central fentanyl conformer of the most populated cluster. The 3D coordinates of the central fentanyl conformer and the corresponding MOR were extracted from the relevant trajectory frames as a reference for further calculations.

### Step 2: Structure preparation

**Fig. 3:**
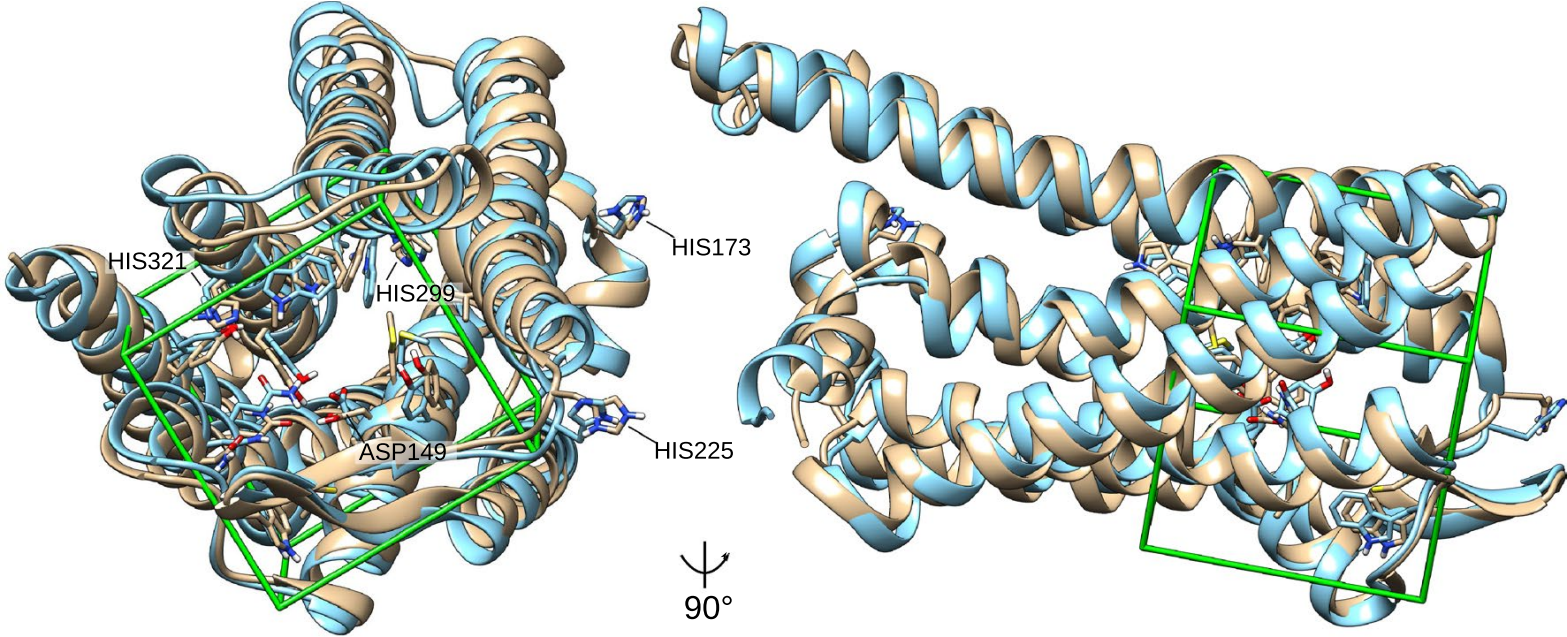
MOR structures for pH-specific docking and target area. Overlay of MOR target structures in neutral (blue) and protonated (beige) state. Side chain protonation states at pH 7.4 and 5 were generated according to $\hbox {p}K_{\hbox {a}}$ values determined by PROPKA, respectively. Side chains in the binding cavity as well as histidine imidazole side chains are depicted. While the imidazole side chains of HIS173 and HIS225 are protonated at acidic pH, they remain neutral in the more buried HIS299 and HIS321. Additionally, the differentially protonated side chain of ASP149 in the binding cavity is shown. The docking target region (“Gridbox”, green lines) was selected based on the fentanyl binding site in the experimental structure (PDB: 8EF5). The left panel shows the MOR from the extracellular angle, and the right panel from the side (90 degrees rotated)

Extracted structures from the trajectory (for the acidic scenario) or the native, experimental structure (for the neutral scenario) in the PDB file format were prepared for molecular docking using PyMOL [41] and AutoDockTools [42]. First, the ligand (fentanyl) and all water molecules were removed from the structures, non-polar hydrogens were removed and each of the structures was converted into PDBQT format. The neutral and acidic MOR structures were then aligned using the PyMOL align function to transfer both receptors into the same coordinate system. Using the aligned structures, a common search space for Autodock Vina [43] scoring functions (“Gridbox”) was designed using AutoDockTools [42]. Therefore, a cuboid box with the size of 20 x 20 x 20 Å was centered on the position of the fentanyl binding site (Fig. 3).

### Step 3: Ligand preparation

To identify novel structural analogs of morphine and fentanyl with pH-specific binding to the MOR, we selected candidate molecules from the CHEMBL database [44, 45]. Therefore, we searched the database with the keywords “fentanyl” and “morphine” and extracted all molecules reported by the search engine. This resulted in the selection of 43 unique morphine- and fentanyl-related molecules. For validation of the approach, a fentanyl derivative previously shown to exhibit pH-specificity to the MOR at low pH (NFEPP) [46], six recently reported $\beta$-fluorofentanyls [47–49], and a dissected morphine analog along with its fluorinated derivatives [50] were also added to the library. This focused library of morphine and fentanyl-related structures in total consisted of 55 molecules (ligand library A). As a larger chemical library of previously unknown MOR agonists, we selected the Enamine GPCR library, which consists of 54.080 drug-like molecules [51] (ligand library B). To prepare the ligands for docking at neutral and acidic pH, we created a database of all ligands with each molecule’s SMILES (Fig. 2, Ligand Libraries). We then used VirtualFlow for Ligand Preparation (VFLP) [21] with the chemical toolbox Open Babel [52] to perform desalting and neutralization on the input molecules followed by protonation of the resulting SMILES at a neutral pH of 7.4 and at an acidic pH of 5.0 using Chemaxon (https://www.chemaxon.com). For the reported fluorinated fentanyl and morphine derivatives, their previously determined $\hbox {p}K_{\hbox {a}}$ values [38, 47, 50] were used to obtain the protonation states, respectively. For all other molecules, $\hbox {p}K_{\hbox {a}}$ values and protonation states were calculated using Chemaxon. Finally, the conformer generation functionality of VFLP and Chemaxon was used to generate the 3D representations of the non-protonated and protonated molecules in the PDBQT format (Fig. 2, Ligand Preparation).

### Step 4: Docking

Binding free energy calculations were performed using VirtualFlow for Virtual Screening (VFVS) [21] on a high-performance computing cluster using the faster Autodock Vina [43] implementation Quick Vina 2 [53]. The docking target region was selected as described above. For each pH scenario, three or six independent docking runs were performed for each ligand-target combination and the exhaustiveness of the docking program was set to 5. Consequently, in the docking runs, the ligands prepared at pH 7.4 are docked to the neutral environment MOR (“neutral pH scenario”), and the ligands prepared at pH 5.0 to the target region of the acidic environment MOR with differentially protonated side chains (“acidic pH scenario”).

### Step 5: Analysis

**Fig. 4:**
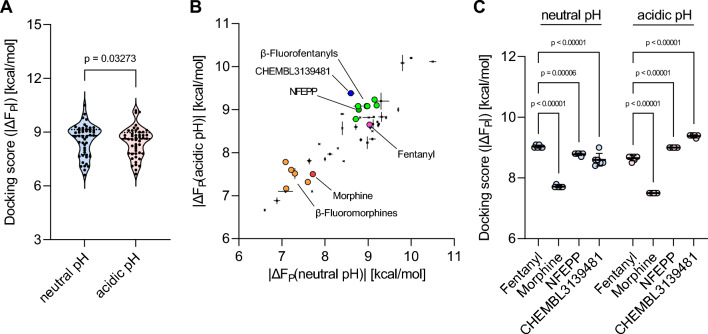
Results of differential docking of morphine- and fentanyl-related ligands. **A** Distribution of docking scores, i.e., binding free energies $\Delta F_P(\text {pH}= 7.4)$ and $\Delta F_P(\text {pH}=5.0)$, for ligand library A at neutral and acidic pH. Paired t-test, two-tailed. **B** Docking scores of morphine- and fentanyl-related molecules to the MOR at neutral (x-axis) plotted against docking scores to the MOR at acidic pH (y-axis). Reference compounds [morphine (red), fentanyl (magenta), NFEPP (green), $\beta$-fluorofentanyls (neon green), $\beta$-fluoromorphines (orange)] and pH-specific hit compound [CHEMBL3139481 (blue)] are highlighted. Data points indicate mean docking scores, horizontal lines indicate standard error of the mean (SEM) of neutral pH scores, and vertical lines SEM of acidic pH scores. **C** Comparison of the MOR docking scores of fentanyl, NFEPP, morphine and CHEMBL3139481 obtained under neutral and acidic pH conditions. Individual data points show scores of replicate dockings, horizontal bars indicate mean values, and error bars indicate standard deviations (SD). Mean docking scores were analyzed by two-way ANOVA with Šídák’s multiple comparisons test

Results of binding free energy calculations were analyzed and plotted using GraphPad Prism (version 9). To exclude docking scores from failed runs, systematic outlier elimination was performed using the ROUT method [54] before the mean docking scores in kcal/mol and standard errors of the mean were calculated. Docking scores for all ligands obtained at neutral pH were compared to docking scores obtained at acidic pH by an unpaired, two-tailed t-test. Docking scores of top hits were compared to fentanyl using a two-way ANOVA with Šídák’s multiple comparisons test (Figs. 4A, 6A).

### Step 6: Optimization

Step 5 results in the trimmed means $|\Delta F_p(L(\theta _i),T(\theta _i)|$, $i=1,2$, of the binding free energies for all ligand-target combinations for $\theta _1=$ pH 5.0 and $\theta _2=$ pH 7.4. Given these values, the multi-objective optimization problem (6) can be solved by simple enumeration for all $\lambda \in [0,1]$, resulting in a group of ligands that all are part of the Pareto front for the results of the ligand library under consideration.

### Utilizing the differential docking pipeline

There are mainly three different strategies for using the pipeline:

- Find promising pH-specific ligands in a relatively *small* set of ligands that have been *pre-selected* because of their structural similarity to known agonists for the target under consideration. For such small ligand libraries, the aim will be to identify the ligands with the best pH-selectivity and compare them to ones with known effects; the set of all Pareto-optimal ligands is of less importance. We will illustrate this case based on the ligand library A.
- Select promising ligands from a *large* library of candidate molecules. In this case, the high-throughput screening option of our differential docking pipeline is required for computing the set of Pareto-optimal ligands that contains the most promising candidate molecules. We will illustrate this case based on the ligand library B.
- Explore the set of all chemically possible ligands (“chemical space”) in order to identify the Pareto front (the set of all Pareto-optimal ligands) without restricting the search to a pre-selected ligand library. Because of the immense size of the chemical space this task requires the development of novel search methods and will not be considered further herein.

## Results and discussion

Next, we present the results of the proposed multi-objective optimal affinity approach in application to the case of MOR-agonists at acidic and neutral pH. The results will first be discussed for our small ligand library A and then for the high-throughput case for library B.

### Identifying pH-specific morphine- and fentanyl-related ligands

We first analyzed the overall docking scores of the neutral and acidic MOR docking results from library A, the morphine- and fentanyl-related molecules selected from the CHEMBL library and the previously reported fluorinated fentanyl and morphine derivatives. Comparing the distribution of the docking scores between the neutral and acidic scenarios, we observed slightly increased average scores for the neutral pH MOR compared to the acidic pH MOR docking (Fig. 4A, p = 0.03273). However, for both docking scenarios we identified morphine and fentanyl analogs with docking scores $|\Delta F_P| > 9\;kcal/mol$ (Fig. 4A), which indicates rather strong predicted binding affinities.

We plotted the docking scores at neutral pH (x-axis) against the docking scores at acidic pH (y-axis) (Fig. 4B). While the molecules to the very right (strongest predicted binding affinity at neutral pH) would be identified and potentially prioritized by most virtual (and experimental) screenings, the molecules with preferential binding at acidic pH are located on the upper left side of the distribution of docking scores, (Fig. 1). Interestingly, we found that NFEPP, a fentanyl derivative, which was previously shown to preferentially bind the MOR at acidic pH and cause fewer side-effects *in vivo* [38], is predicted to bind to the MOR at neutral pH with lower affinity as fentanyl (8.8 vs. 9.0 kcal/mol; p = 0.00006), but to bind the MOR at acidic pH with significantly higher affinity (9.0 vs. 8.7 kcal/mol; p < 0.00001) (Fig. 4C). For the $\beta$-fluorofentanyls that were recently reported to have an increased potency at acidic pH compared to fentanyl [47], we also found increased predicted binding affinities at acidic pH and similar, slightly higher or even lower predicted binding affinities at neutral pH (Fig. 4B, $\beta$-fluorofentanyls). Similar data were obtained for morphine and the recently reported $\beta$-fluoromorphines [50]: several fluorinated morphines are located to the upper left of morphine indicating an increased preference for the MOR at acidic pH (Fig. 4B, $\beta$-fluoromorphines). Interestingly, within the ChEMBL molecules of morphine- and fentanyl-related structures, the morphine analog CHEMBL3139481 strongly separated from the distribution of the scores and demonstrated the highest preference for the MOR at low pH compared to morphine, fentanyl and also NFEPP (Fig. 4B). Compared to fentanyl, this morphine-related molecule showed a reduced predicted binding affinity to the neutral MOR (8.6 vs. 9.0 kcal/mol, p < 0.00001), and a strongly increased predicted binding affinity to the acidic MOR (9.4 vs. 8.7 kcal/mol; p < 0.00001) (Fig. 4C).

**Fig. 5:**
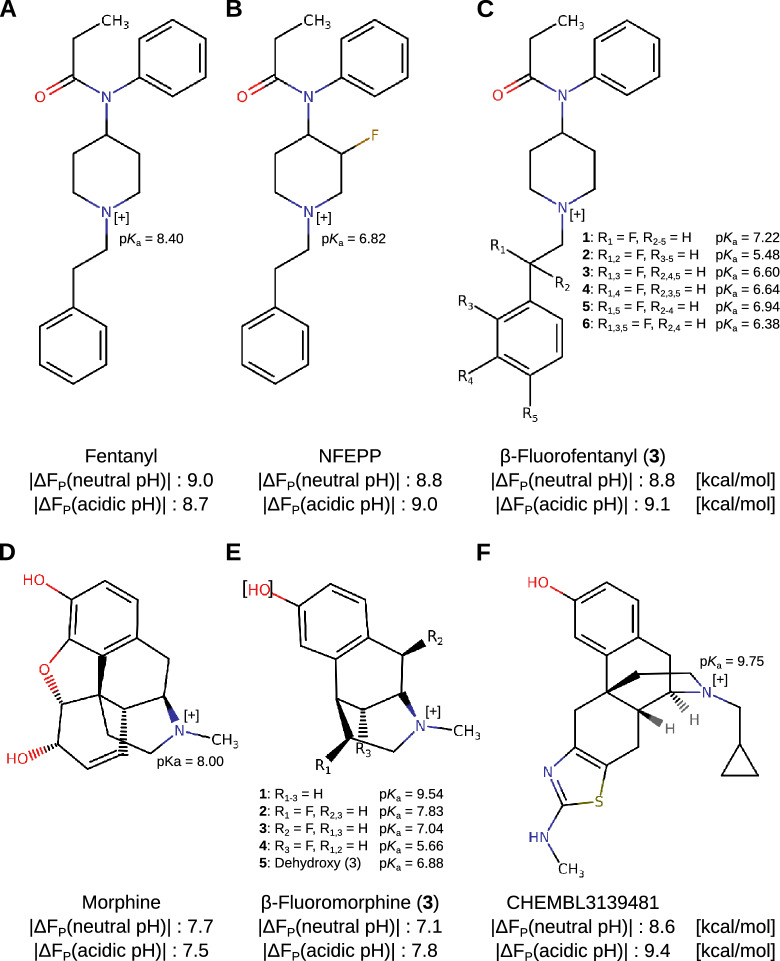
Chemical structures of the highlighted molecules from library A: Chemical structures of fentanyl (**A**), NFEPP (**B**), $\beta$-fluorofentanyls (**C**), morphine (**D**), $\beta$-fluoromorphines (**E**) and CHEMBL3139481 (**F**). Docking scores in kcal/mol of neutral or acidic MOR docking scenarios are indicated below the structures, respectively. For the $\beta$-fluorofentanyls and -morphines the different screened derivatives are indicated by numbers 1–6 or 1–5, respectively. The docking scores of the derivative with the strongest pH specificity are shown. The potentially differently protonated nitrogen atoms in the compound structures are marked with [+]. The $\hbox {p}K_{\hbox {a}}$ values of fentanyl, morphine and its fluorinated derivatives are indicated as previously described [47, 50] and the experimentally determined value is shown, if available. For CHEMBL3139481, the calculated estimate of its $\hbox {p}K_{\hbox {a}}$ value is shown

We next analyzed the chemical structures of fentanyl, NFEPP, morphine, their fluorinated derivatives, and the CHEMBL3139481 molecule (Fig. 5). Compared to fentanyl (Fig. 5A), NFEPP carries an additional fluorine on the piperidine moiety (Fig. 5B), which represents a strong electron-withdrawing group and can influence the protonation state of the piperidine ring structure. The recently reported group of $\beta$-fluorofentanyls are designed similarly: substitution of hydrogen with fluorine at various positions also influences the protonation state of the nitrogen in the piperidine ring structure. Notably, the $\beta$-fluorofentanyl named RR-49 (or 12a), which experimentally showed the strongest preference for the MOR in an acidic environment [47], also demonstrated the strongest pH specificity in our binding affinity predictions (Fig. 5C, $\beta$-fluorofentanyl 3). Additionally, the binding affinity estimations obtained for morphine (Fig. 5D) compared to $\beta$-fluoromorphines (Fig. 5E) also confirm previous results: the morphine analog Fluoromorphine $\upbeta$-C2, which previously demonstrated the strongest pH selectivity [50], also showed the highest predicted binding affinity to the acidic MOR over its affinity to the neutral MOR (7.8 vs. 7.1 kcal/mol). Interestingly, the molecule CHEMBL3139481 (Fig. 5F), which overall exhibited the strongest pH specificity, belongs to a group of aminothiazolomorphinans, a group of morphine-like opioids previously shown to be a potential pharmacotherapeutic approach to reduce drug abuse [55, 56]. CHEMBL3139481 (or MCL-742) was previously reported to bind with sub-nanomolar affinity to all three, the MOR, the $\kappa$-opioid receptor (KOR), and the $\delta$-opioid receptor (DOR) [57]. Interestingly, in comparison to morphine (Fig. 5D), it also harbors an electron-withdrawing moiety (cyclopropanyl) close to a carbon-nitrogen ring structure (Fig. 5F), which could exhibit a similar effect on CHEMBL3139481’s nitrogen ring structure as the fluorine in fluorofentanyls on the piperidine moiety. Our results suggest a potential pH-specificity of this morphine analog, which should be further investigated in experimental studies.

### Pareto-optimal pH-specific ligands in the Enamine GPCR library

**Fig. 6:**
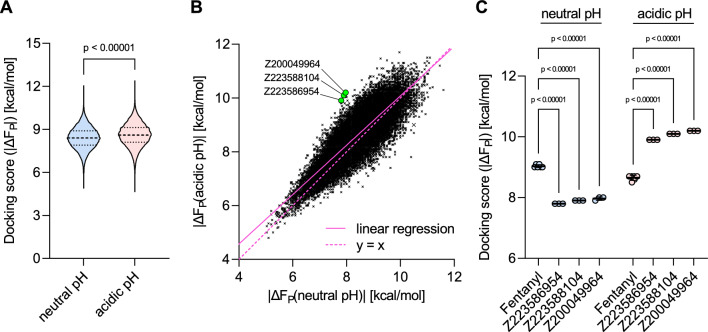
Results of differential docking of ligand library B, extracted from the Enamine GPCR library. **A** Distribution of docking scores at neutral and acidic pH, binding free energies $\Delta F_P(\text {pH}= 7.4)$ and $\Delta F_P(\text {pH}=5.0)$, respectively. Paired t-test, two-tailed. **B** Docking scores of ligands to the MOR at neutral (x-axis) are plotted against docking scores to the MOR at acidic pH (y-axis). The best acidic pH-specific hit molecules are highlighted [Z223586954, Z223588104, Z200049964 (neon green)]. Individual data points in **C** show scores of replicate dockings, horizontal bars indicate mean values, and error bars indicate standard deviations (SD). Mean docking scores were analyzed by two-way ANOVA with Šídák’s multiple comparisons test

**Fig. 7:**
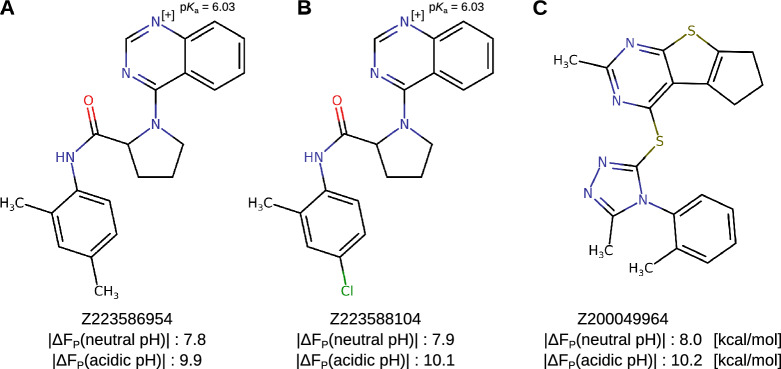
Chemical structures of the highlighted molecules with the highest absolute value of the difference between its acidic and neutral pH docking scores, and a predicted binding affinity of $\le$ 8 kcal/mol at neutral pH (green dots in Fig. 6B) from library B. The molecules Z223586954 (**A**) and Z223588104 (**B**) are predicted to be protonated at pH 5.0 but to remain neutral at pH 7.4. Z223588104 (**B**) and Z200049964 (**C**) show the highest predicted binding affinity difference between the acidic and the neutral MOR docking scenarios. Docking scores of neutral or acidic MOR docking scenarios are indicated below the structures and calculated $\hbox {p}K_{\hbox {a}}$ values of the ionisable atoms are shown, respectively

In contrast to rational drug design based on a small set of known chemical structures, our differential docking pipeline can also be used for *ab initio* drug discovery. In order to enable this, we have implemented the method in VirtualFlow, which is capable of performing ultra-large virtual screens in a high-throughput manner [21]. For an illustration of a multi-objective affinity approach to the identification of previously unknown chemical structures with pH-specific binding to the MOR, we applied our differential docking pipeline to over 50K ligands from the Enamine GPCR Library. This library consists of drug-like molecules readily available at Enamine. Similarly as for the morphine- and fentanyl-related molecules, we performed neutral and acidic pH docking runs to the MOR and analyzed the results. Comparing the distribution of the docking scores between the neutral and acidic scenarios, we observed slightly higher maximal scores for the acidic pH MOR compared to the neutral pH MOR docking (Fig. 6A, 8.6. vs. 8.4 kcal/mol, p < 0.00001). However, we found a similar range of docking scores ($|\Delta F_P|$) for both scenarios, ranging from 11.6 to 5.0 kcal/mol for the neutral and 11.7 and 4.8 kcal/mol for acidic pH docking scenarios, respectively. To identify acidic pH-specific binders, we again plotted the docking scores at neutral pH against the docking scores at acidic pH (Fig. 6B). The molecules with higher predicted binding affinities at acidic compared to neutral pH are located to the upper left of the bisector ($y = x$ in Fig. 6B). The top three molecules with a docking score at neutral pH $\le$ 8 kcal/mol and with the highest absolute value of the difference between its acidic and neutral pH docking scores, i.e., the optimal ligands from (6) for $\lambda \approx 1/2$, are the ones with labels Z223586954, Z223588104, and Z200049964; these are highlighted in Fig. 6B (green dots), while their chemical structures are shown in Fig. 7. The selected hits from the Enamine GPCR Library each have a lower predicted binding affinity to the neutral pH MOR, but a significantly higher predicted binding affinity to the acidic pH MOR compared to fentanyl (Fig. 6C). Thus, these molecules represent interesting candidates for further investigation in identifying pH-specific MOR agonists. Interestingly, similarly to fluorinated fentanyl and morphine derivatives, two of the molecules are also predicted to be protonated at pH 5.0 but to remain neutral at pH 7.4 (Fig. 7A, B). Strikingly, the top two molecules (Fig. 7B, C) show a predicted binding affinity difference of 2.2 kcal/mol between the acidic and the neutral MOR docking scenario indicating an even stronger pH specificity than previously identified ligands.

**Fig. 8:**
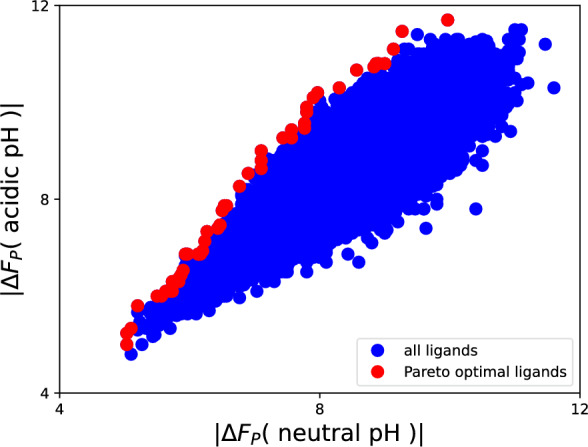
Pareto-optimal ligands (red) of all successfully screened 50.838 molecules from the Enamine GPCR library B (blue)

Generally, when solving the multi-objective optimization problem to identify pH-specific MOR ligands (6) for all $\lambda \in [0,1]$, we get the Pareto-optimal ligands shown in Fig. 8, among them the three structures mentioned above. The size of the Pareto set can also be varied: the Pareto set can be extended by instead of only using the solutions (6) of the optimization problem, as shown in Fig. 1 by the red line and in Fig. 8 by the red dots, one might also take the second best, third best and so on. In Fig. 1 this would result in a thicker red line. We call this the $\varepsilon$-Pareto front. Alternatively, the Pareto set can be reduced: In addition to the desired pH specificity, which the molecules of the Pareto set fulfill, care must be taken that the overall binding affinity to the target in its desired environment is not too low. This can be achieved by setting a threshold $\alpha$ for $\Delta F_P$ such that $|\Delta F_P|\ge \alpha$. Such a threshold can also be included in the multi-objective framework as an additional constraint. This flexibility of the Pareto set can be used as a starting point for further global optimization strategies in the chemical space to identify specific ligands with desired properties.

## Conclusion

In most virtual screening methods, binding affinity to the target structure is the decisive measure. Accordingly, the search in the chemical space is for ligands that have the highest binding affinity. The strength of the binding affinity can be described with the help of scoring functions, such that the ligand with the best score and thus best binding affinity is the result of an optimization problem. In the new approach presented herein, however, we do not only consider the binding affinity but also the chemical environment of the protein-ligand system in the search for the optimal ligand which leads to a different search strategy in the chemical space. This new strategy takes the form of a multi-objective optimization problem. In the case where the discriminative effect of the environment can be characterized by a single parameter $\theta$ with desired effects for a value $\theta _1$ and unwanted one for $\theta _2$. In this scenario, the objective is to maximize the binding affinity for $\theta _1$ while minimizing it for $\theta _2$.

Using the $\mu$-opioid receptor as an example, we could already show that taking pH as a discriminative parameter with $\theta _1=\text {pH}\ 5.0$ and $\theta _2=\text {pH}\ 7.4$, side effects can be taken into account when searching for an optimal ligand. This lead to the identification of a fentanyl derivative (N-(3-fluoro-1-phenethylpiperidin-4-yl)-N-phenylpropionamide, NFEPP) with a similar analgesic efficacy as fentanyl but less side effects *in vivo* [38] and the design of additional fluorinated fentanyl and morphine derivatives [47–50]. If our virtual screening pipeline was built on the traditional optimal affinity approach, it would aim to maximize binding affinity to the $\mu$-opioid receptor (MOR) and would prefer fentanyl over NFEPP, because fentanyl has a higher binding affinity at neutral pH than NFEPP (it is more right in Fig. 4B). However, NFEPP shows fewer side effects than fentanyl [38]. The conventional approach would prefer fentanyl also to morphine and other molecules only due to its higher binding affinity. In our new approach fentanyl is considered “worse” than NFEPP since it shows a higher binding affinity to the neutral MOR and a lower affinity to the acidic MOR.

The results obtained here using a multi-objective approach also seem to support a recent chemical idea of how to improve opioids for showing less side effects. In order to see this, let us compare the pair fentanyl/NFEPP: the difference between the two structures is just given by replacing a hydrogen atom with a fluorine atom in a “two C-atoms”-distance to the nitrogen atom (N) in the piperidine moiety. This N-atom can either be protonated or deprotonated according to the pH value of the environment. By the inductive effect (-I) of the fluorine atom in NFEPP, the electron density at the nitrogen atom decreases compared to fentanyl. This reduces the ability of the N-atom to be protonated, which is important for efficient binding to the MOR. Thus, only in inflamed tissue (at low pH) NFEPP is mainly protonated and active, while fentanyl is active at low pH in inflamed but also at neutral pH in healthy tissue. The same principal was also applied to generate additional fluorinated fentanyl and morphine derivatives that, at least in part, have demonstrated similar pH specificity to the MOR as NFEPP ([47, 50]). Interestingly, the pair morphine/CHEMBL3139481 in Fig. 5 (structures D and F) is of a similar kind: The chemical scaffold in which the nitrogen atom is placed is extended by an electron-withdrawing moiety (cyclopropanyl) located at a “two C-atoms”-distance from the nitrogen. In our differential docking approach aiming at pH-specificity the morphine analog CHEMBL3139481 was identified and is considered a pH-specific, “better” than morphine, and even fentanyl and NFEPP. It would be interesting to further test this substance in laboratory experiments, because it is one of the “best” candidates in the examined set of opioids from the CHEMBL library. This substance may also be favorable for clinical use, because it is a morphine-related molecule but not a derivative of fentanyl, which is considered to be a major cause of the opioid crisis [58].

As one of the basic steps of the computational experiment presented herein, we generated neutral and acidic environment MOR structures by neutralization or protonation of its side-chains. While we used the experimentally determined conformation of the MOR for the neutral docking scenario, we performed subsequent MD simulation of the acidic protonation state so that the differential protonation of the amino acid side chains can exert its effect on the conformation. Additionally, we prepared ligands for pH-specific docking by predicting their protonation state in a neutral or acidic environment. While on the one hand this approach can yield pH-specific ligands by changes in binding affinity due to direct interactions between the (neutral or protonated) ligand and differentially protonated side-chains, also the protonation state-related conformational changes to the binding cavity can affect pH-specific binding. It is important to note, however, that prediction of both the $\hbox {p}K_{\hbox {a}}$ of small molecule ligands and amino acid side chains in large macromolecules is challenging and may be error-prone. Thus, our results rely on experimental validation but can serve as an early prioritization strategy to identify pH-specific binders.

The multi-objective optimal affinity approach and its implementation via a differential docking pipeline are general in the sense that can be utilized independent from the application to finding pH-specific agonists for the MOR presented. It will apply whenever drug design is based on several partially conflicting objectives. These objectives may result from desiring different ligand-target interactions at different environmental conditions (as discussed herein) but can also be used to describe other conflicting aims like maximizing binding affinity versus minimizing side effects or toxicity e.g. associated with binding to other targets, for example. Additionally, a multi-objective optimal affinity approach can serve as a drug discovery pipeline in the search for dual inhibitors or polypharmacological molecules, in which it is the goal to identify drugs that bind multiple targets with high affinity [59, 60]. By modifying the corresponding optimization problem accordingly (6), our method is also able to seek for such multivalent structures.

In current drug discovery, most molecules identified in primary screenings and prioritized after lead optimization studies fail at a later stage in the drug development process. One of the main reasons for this high drop-out rate are safety issues at the preclinical or even clinical stage, putting a huge financial risk on drug development programs. Thus, early identification of chemical compounds and subclasses at risk to cause unwanted and severe side effects is key to prevent such extremely expensive pitfalls. By performing multi-objective optimal affinity optimization in virtual screening approaches, i.e. prioritizing molecules with a high binding affinity for the target structure(s) in the desired, diseased environment, while deprioritizing molecules with high binding affinity for the target structure(s) in the undesired, healthy environment, the selection of candidate molecules with a good safety profile can already be implemented at the initial screening process. With the expected increase in computing power and the development of faster search algorithms in predicting binding affinities, this approach can be further extended to multiple targets, e.g. key regulatory proteins or enzymes of essential cellular processes known to bear safety concerns. Ultimately, performing multi-objective affinity optimization in virtual drug discovery should contribute to the identification and development of safer, more specific drugs at higher pace.

## Data Availability

All docking data and the prepared MOR target structures are available via https://www.zib.de/ext-data/pH_specific_screening_MOR/. The code that was used to conduct the ligand preparations and virtual screening is available at https://github.com/VirtualFlow/VFLP and https://github.com/VirtualFlow/VFVS.

## References

[1] Schmitz R (1985). Friedrich Wilhelm Serturner and the discovery of morphine. Pharm Hist.

[2] Sneader W (1998). The discovery of heroin. Lancet.

[3] Lionta E, Spyrou G, Vassilatis D, Cournia Z (2014). Structure-based virtual screening for drug discovery: principles, applications and recent advances. Curr Top Med Chem.

[4] Bhunia SS, Saxena M, Saxena AK, Saxena AK (2021). Ligand- and structure-based virtual screening in drug discovery. Biophysical and Computational Tools in Drug Discovery.

[5] Korn M, Ehrt C, Ruggiu F, Gastreich M, Rarey M (2023). Navigating large chemical spaces in early-phase drug discovery. Curr Opin Struct Biol.

[6] Medina-Franco JL, Chavez-Hernandez AL, Lopez-Lopez E, Saldivar-Gonzalez FI (2022). Chemical multiverse: an expanded view of chemical space. Mol Inform.

[7] Bohacek RS, McMartin C, Guida WC (1996). The art and practice of structure-based drug design: a molecular modeling perspective. Med Res Rev.

[8] Li Q (2020). Application of fragment-based drug discovery to versatile targets. Front Mol Biosci.

[9] St JD, Denis RJ, Hall CW, Murray TD, Heightman Rees DC (2021). Fragment-based drug discovery: opportunities for organic synthesis. RSC Med Chem.

[10] Rarey M, Kramer B, Lengauer T, Klebe G (1996). A fast flexible docking method using an incremental construction algorithm. J Mol Biol.

[11] Ewing TJ, Makino S, Skillman AG, Kuntz ID (2001). DOCK 4.0: search strategies for automated molecular docking of flexible molecule databases. J Comput Aided Mol Des.

[12] Foerster JM, Poehner I, Ullmann GM (2018). Mcmap–a computational tool for mapping energy landscapes of transient protein-protein interactions. ACS Omega.

[13] Korb O, Stützle T, Exner TE (2007). An ant colony optimization approach to flexible protein-ligand docking. Swarm Intell.

[14] Jones G, Willett P, Glen RC, Leach AR, Taylor R (1997). Development and validation of a genetic algorithm for flexible docking edited by f. e. cohen. J Mol Biol.

[15] DEA Fentanyl awareness (2022) https://www.dea.gov/fentanylawareness. Accessed 1 Dec 2023

[16] Sinha S, Mitra R, Pal S (2008). Temperature-dependent simultaneous ligand binding in human serum albumin. J Phys Chem B.

[17] Papaneophytou CP, Grigoroudis AI, McInnes C, Kontopidis G (2014). Quantification of the effects of ionic strength, viscosity, and hydrophobicity on protein-ligand binding affinity. ACS Med Chem Lett.

[18] Kuhn HW, Tucker AW (1951) Nonlinear programming. In: Proceedings of the Second Berke ley Symposium on Mathematical Statistics and Probability, Berkeley, Calif.: University of California Press, pp. 481-492. https://projecteuclid.org/euclid.bsmsp/1200500249

[19] Schuetze O, Hernandez Castellanos C (2021). Archiving strategies for evolutionary multi-objective optimization algorithms.

[20] Olsson MHM, Søndergaard CR, Rostkowski M, Jensen JH (2011). Propka3: consistent treatment of internal and surface residues in empirical $$\text{p}K_{\text{ a }}$$ predictions. J Chem Theory Comput.

[21] Gorgulla C (2020). An open-source drug discovery platform enables ultra-large virtual screens. Nature.

[22] wwPDB consortium (2019). Protein data bank: the single global archive for 3d macromolecular structure data. Nucleic Acids Res.

[23] Zhuang Y (2022). Molecular recognition of morphine and fentanyl by the human $$\mu$$-opioid receptor. Cell.

[24] Sondergaard CR, Olsson MHM, Rostkowski M, Jensen JH (2011). Improved treatment of ligands and coupling effects in empirical calculation and rationalization of $$\text{ p }K_{\text{ a }}$$ values. J Chem Theory Comput.

[25] Unni S (2011). Web servers and services for electrostatics calculations with apbs and pdb2pqr. J Comput Chem.

[26] Kim S, Lee J, Jo S, Brooks CL, Lee HS, Im W (2017). Charmm-gui ligand reader and modeler for charmm force field generation of small molecules. J Comput Chem.

[27] Lee J (2016). Charmm-gui input generator for namd, gromacs, amber, openmm, and charmm/openmm simulations using the charmm36 additive force field. J Chem Theory Comput.

[28] Abraham MJ (2015). Gromacs: high performance molecular simulations through multi-level parallelism from laptops to supercomputers. SoftwareX.

[29] Gutierrez IS (2016). Parametrization of halogen bonds in the CHARMM general force field: improved treatment of ligand-protein interactions. Bioorg Med Chem.

[30] Huang J (2017). CHARMM36m: an improved force field for folded and intrinsically disordered proteins. Nat Methods.

[31] Klauda JB (2010). Update of the CHARMM all-atom additive force field for lipids: validation on six lipid types. J Phys Chem B.

[32] Jorgensen WL, Chandrasekhar J, Madura JD, Impey RW, Klein ML (1983). Comparison of simple potential functions for simulating liquid water. J Chem Phys.

[33] Essmann U, Perera L, Berkowitz ML, Darden T, Lee H, Pedersen LG (1995). A smooth particle mesh ewald method. J Chem Phys.

[34] Nosé S (1984). A molecular dynamics method for simulations in the canonical ensemble. Mol Phys.

[35] Hoover WG (1985). Canonical dynamics: equilibrium phase-space distributions. Phys Rev A.

[36] Parrinello M, Rahman A (1981). Polymorphic transitions in single crystals: a new molecular dynamics method. J Appl Phys.

[37] Hess B, Bekker H, Berendsen HJC, Fraaije JGEM (1997). Lincs: a linear constraint solver for molecular simulations. J Comput Chem.

[38] Spahn V (2017). A nontoxic pain killer designed by modeling of pathological receptor conformations. Science.

[39] Ray S, Sunkara V, Schütte C, Weber M (2020). How to calculate pH-dependent binding rates for receptor-ligand systems based on thermodynamic simulations with different binding motifs. Mol Simul.

[40] Daura X, Gademann K, Jaun B, Seebach D, van Gunsteren WF, Mark AE (1999). Peptide folding: when simulation meets experiment. Angewandte Chemie Int Ed.

[41] Schrödinger LLC (2015) The PyMOL molecular graphics system, version 1.8

[42] Sanner MF (1999). Python: a programming language for software integration and development. J Mol Graph Model.

[43] Trott O, Olson AJ (2010). AutoDock Vina: improving the speed and accuracy of docking with a new scoring function, efficient optimization, and multithreading. J Comput Chem.

[44] Gaulton A (2012). ChEMBL: a large-scale bioactivity database for drug discovery. Nucleic Acids Res.

[45] Bento AP (2014). The ChEMBL bioactivity database: an update. Nucleic Acids Res.

[46] Stein C, Weber M, Scharkoi O, Deuflhard P (2013) Method and system for indentifying compounds that bind and/or activate a target opioid receptor in a ph-dependent manner. WO patent, vol. WO2013102681A1

[47] Rosas R, Huang XP, Roth BL, Dockendorff C (2019). Fluorofentanyls are pH-Sensitive mu opioid receptor agonists. ACS Med Chem Lett.

[48] Spahn V (2018). Opioid receptor signaling, analgesic and side effects induced by a computationally designed pH-dependent agonist. Sci Rep.

[49] Del Vecchio G (2019). Of opioid ligands as a discriminating factor for side effects. Sci Rep.

[50] Augenstein M, Alexander N, Gartner M (2023). Computational design and molecular modeling of morphine derivatives for preferential binding in inflamed tissue. Pharmacol Res Perspect.

[51] Enamine GPCR Library designed for discovery of new gpcr ligands, 54 080 compounds, https://enamine.net/compound-libraries/targeted-libraries/gpcr-library. Accessed 10 Feb 2023

[52] O’Boyle NM, Banck M, James CA, Morley C, Vandermeersch T, Hutchison GR (2011). Open babel: an open chemical toolbox. J Cheminform.

[53] Alhossary A, Handoko SD, Mu Y, Kwoh CK (2015). Fast, accurate, and reliable molecular docking with QuickVina 2. Bioinformatics.

[54] Motulsky HJ, Brown RE (2006). Detecting outliers when fitting data with nonlinear regression - a new method based on robust nonlinear regression and the false discovery rate. BMC Bioinform.

[55] Zhang T (2011). Opioid activity. J Med Chem.

[56] Wang YJ (2009). Pharmacological characterization of ATPM [(-)-3-aminothiazolo[5,4-b]-N cyclopropylmethylmorphinan hydrochloride], a novel mixed kappa-agonist and mu-agonist/- antagonist that attenuates morphine antinociceptive tolerance and heroin self-administration behavior. J Pharmacol Exp Ther.

[57] Provencher BA (2013). Synthesis and pharmacological evaluation of aminothiazolomorphi nans at the mu and kappa opioid receptors. J Med Chem.

[58] Jannetto PJ, Helander A, Garg U, Janis GC, Goldberger B, Ketha H (2019). The fentanyl epidemic and evolution of fentanyl analogs in the United States and the European Union. Clin Chem.

[59] Wang Z, Yang B (2022). Polypharmacology.

[60] Ciceri P (2014). Dual kinase-bromodomain inhibitors for rationally designed polypharmacology. Nat Chem Biol.

